# Association of aquaporin-4 antibody-seropositive optic neuritis with vision-related quality of life and depression

**DOI:** 10.3389/fneur.2023.1265170

**Published:** 2023-09-29

**Authors:** Ruitong Song, Wenqiao Huang, Jun Yang, Xueshan Tang, Yihua Huang, Yingying Chen, Mukun Zhao, Qiuming Hu, Yi Du

**Affiliations:** ^1^Department of Ophthalmology, The First Affiliated Hospital of Guangxi Medical University, Nanning, China; ^2^Department of Ophthalmology, Wuzhou Gongren Hospital, Wuzhou, China; ^3^Department of Ophthalmology, Wuming Hospital of Guangxi Medical University, Nanning, China; ^4^Department of Ophthalmology, The Second Affiliated Hospital of Guangxi Medical University, Nanning, China; ^5^Jingliang Eye Hospital, Guangxi Medical University, Nanning, China

**Keywords:** optic neuritis, neuromyelitis optica spectrum disorder, aquaporin-4, vision-related quality of life, depression

## Abstract

**Objective:**

Aquaporin-4 (AQP4) antibody-seropositive optic neuritis (AQP4-ON) is one of the most common types of optic neuritis in China. However, the association between AQP4-ON and vision-related quality of life (QoL) and depression remains poorly understood.

**Methods:**

In this cross-sectional study, 57 patients with optic neuritis were evaluated for their vision-related QoL using a Chinese version of the 25-item National Eye Institute Visual Function Questionnaire (VFQ-25) and assessed for depressive symptoms using a Chinese version of the Beck Depression Inventory-II (BDI-II). Data regarding participants’ age, sex, visual acuity, and the number of recurrence events were gathered. Linear regression analysis was employed to investigate the relationships between AQP4-ON and vision-related QoL, as well as depression.

**Results:**

Of the 57 included patients, 28 were AQP4-ON, and 29 were idiopathic optic neuritis (ION). AQP4-ON demonstrated a significant correlation with a decreased VFQ-25 composite score (Mean difference, −11.65 [95% CI, −21.61 to −1.69]; *p* = 0.023) and an increased BDI-II score (Mean difference, 6.48 [95% CI, 0.25 to 12.71]; *p* = 0.042) when compared to ION. The BDI-II score was correlated with the VFQ-25 composite score (Spearman ρ = −0.469; *p* < 0.001) but not with the visual acuity in the worse-seeing eye (Spearman ρ = 0.024; *p* = 0.860) or in the better-seeing eye (Spearman ρ = −0.039; *p* = 0.775), bilateral severe visual impairment (Spearman ρ = 0.039; *p* = 0.772) or the number of recurrence events (Spearman ρ = 0.184; *p* = 0.171).

**Conclusion:**

AQP4-positive optic neuritis is associated with a decline in vision-related quality of life as well as an increased likelihood of experiencing depression. It is crucial for clinicians to assess both vision-related QoL and depression in patients with AQP4-positive optic neuritis to provide patient-centered care.

## Introduction

Optic neuritis (ON) is an inflammatory disease that affects the optic nerve and can cause visual dysfunction and pain. The landmark Optic Neuritis Treatment Trial (ONTT) study demonstrated that typical optic neuritis significantly impairs patients’ vision-related quality of life (QoL) when compared with healthy individuals (1). Many patients with other eye disorders, such as macular degeneration and glaucoma, experience reduced QoL as well (2).

Visual impairment is associated with a number of adverse psychosocial outcomes, particularly depression (3). The cause of this depression is unclear, but it is generally believed to be the result of an interaction of social, psychological, and biological factors (4). Moreover, it is possible for patients with eye diseases to suffer from depression or depression symptoms due to various ocular symptoms, including pain, discomfort, foreign body sensation, and others (5).

Aquaporin-4 (AQP4) antibody-seropositive optic neuritis (AQP4-ON) is a phenotype of neuromyelitis optica spectrum disorder (NMOSD). Recent studies have demonstrated the significant impact of NMOSD on multiple aspects of quality of life, including an elevated risk of depression (6, 7). In China, 35–50% of optic neuritis patients are AQP4 antibody seropositive (8, 9). However, little is known about the relationship between AQP4-ON and vision-related QoL. This study aimed to investigate the correlation between AQP4-ON and vision-related QoL as well as depression.

## Methods

### Participants

We conducted a multicenter, cross-sectional study from June 2016 to August 2020 to evaluate optic neuritis patients’ vision-related quality of life and depression in 4 tertiary hospitals in the Guangxi Zhuang Autonomous Region, China. Patients were included if (1) they were aged greater than or equal to 18 years, (2) they were able to complete the questionnaire independently or with the assistance of another person and (3) their serum AQP4-IgG antibody status was available. They were excluded if (1) they had ocular diseases affecting vision (e.g., glaucoma, cataract), (2) they had a history of ocular surgery, and (3) they had clinical evidence of ischemia, inherited disorders, compression, or intoxication causing optic neuropathy. Serum AQP4-IgG antibody status was measured using a commercially available cell-based assay (Kindstar Global Ltd. and KingMed Diagnostics Ltd., China). Patients with AQP4-IgG seropositivity were classified as having AQP4-ON. Patients who tested negative for AQP4-IgG and showed no signs of multiple sclerosis, neuromyelitis optica, autoimmune diseases like Sjögren syndrome, or any other identifiable causes were diagnosed with idiopathic optic neuritis (ION).

The Ethics Committee of the First Affiliated Hospital of Guangxi Medical University approved this study, which followed the Helsinki Declaration [No. 2016 (KY-GUOJI-183)]. Patients were given informed consent to participate and complete the self-administered QoL questionnaire and a depression screening questionnaire.

Data was collected pertaining to age, sex, visual acuity, and the number of recurrence events. Visual acuity was assessed using the standardized logarithmic visual acuity chart, a tumbling E acuity chart, at a distance of 5 meters (10). All data collection, questionnaire completion, and serum antibody detection were conducted upon admission, prior to treatment initiation, during the acute attack.

### Assessment of vision-related quality of life

For assessing quality of life, we used a validated Chinese version of the 25-item National Eye Institute Visual Function Questionnaire (VFQ-25) (11). The VFQ-25 contains 11 vision-related scales based on 25 questions that have been validated in the Chinese population regarding vision or feelings about a visual condition. Subscales include general health, general vision, ocular pain, near vision, distance vision, color vision, peripheral vision, social functioning, mental health, role difficulties, and dependency. The composite score ranges from 0 to 100, with a higher score representing better visual function and well-being.

### Assessment of depression

For assessing depressive symptoms, we administered a validated Chinese version of the Beck Depression Inventory II (BDI-II) (12). It contains 21 items with an option from 0 to 3 in each question. The total score is between 0 and 63, with greater scores indicating higher levels of depression. Depressive symptoms can be divided into four categories based on their total score: severe depression, with a score of 29 or higher; moderate depression, with a score of 20 to 28; mild depression, with a score of 14 to 19; and no depression, with a score of 13 or lower.

### Statistical analysis

Demographics, VFQ-25 scores, and BDI-II scores are summarized as the means with standard deviations (SDs) or as frequencies with percentages, when appropriate. Student’s t test or the Mann–Whitney U test was performed on continuous variables to compare differences between two groups, as appropriate. A chi-squared test or Fisher’s exact test was used to analyze categorical data. Linear regression analysis was utilized to investigate the associations between AQP4-ON and vision-related QoL, as well as depression. Correlations between the BDI-II score and the vision-related QoL score and clinical characteristics were calculated using Spearman’s rank correlation (presented as Spearman ρ). For statistical purposes, decimal visual acuity was converted into logarithm of the minimum angle of resolution (logMAR) units. A logMAR value of 1.85, 2.3, 2.6, and 2.9 were assigned for counting fingers, hand motion, light perception, and no light perception, respectively (13). Severe visual impairment was defined as visual acuity of 1.0 logMAR (Snellen equivalent, 20/200) or worse. All statistical analyses were performed using R, version 4.2.2 (R Foundation for Statistical Computing, Vienna, Austria). Statistical significance was considered to be *p* < 0.05 (two-tailed).

## Results

### Demographic and clinical characteristics

**Figure 1:**
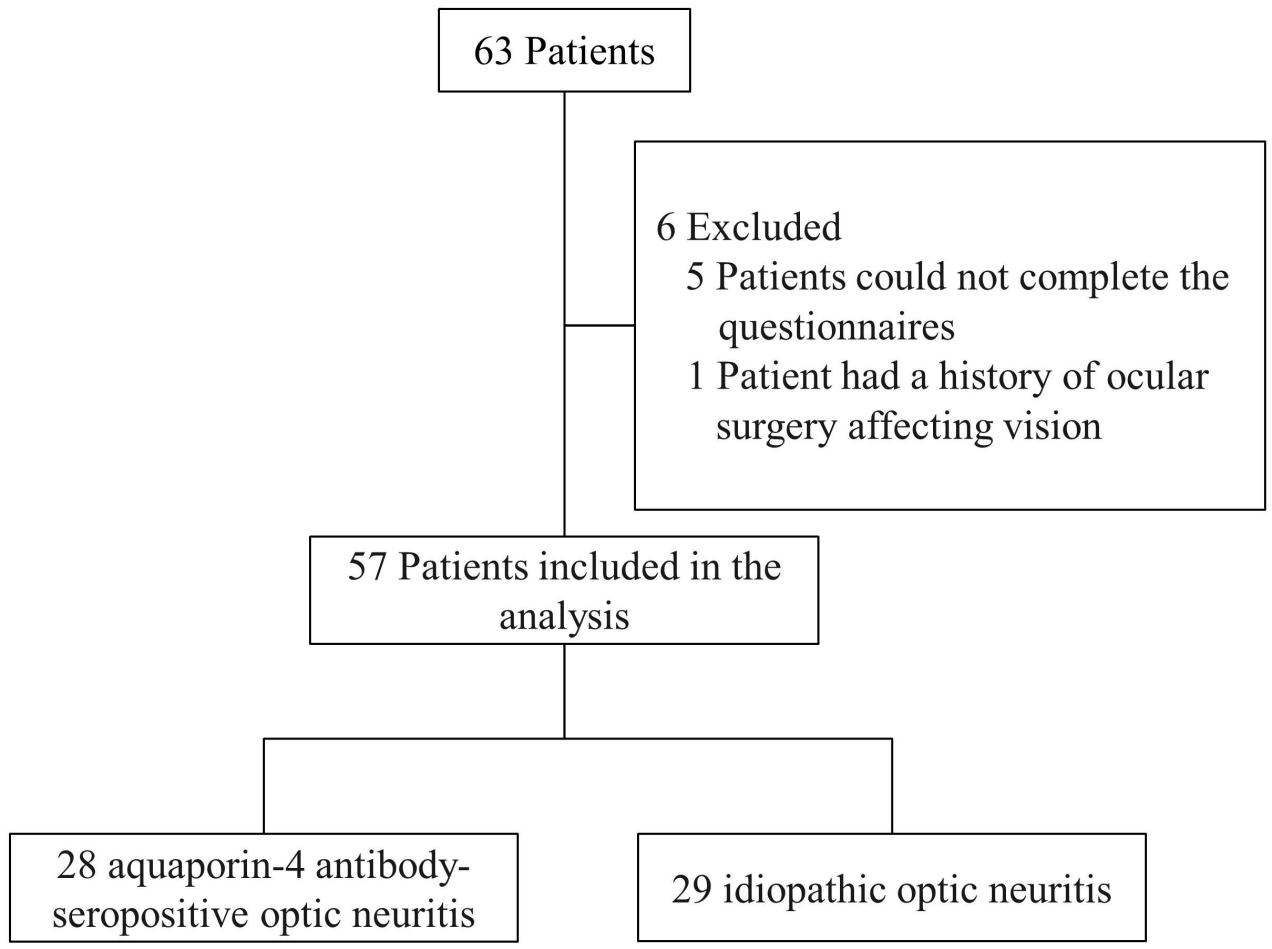
Study flowchart.

In this study, 28 patients (49.1%) with AQP4-ON and 29 patients (50.9%) with idiopathic ON who completed the VFQ-25 and the BDI-II were included for analysis (Figure 1). A summary of patients enrolled at the 4 study sites is available in Supplementary Table. The mean age was 38.84 years (SD, 14.88 years), and 68.4% (39/57) of the patients were female. The median visual acuity was counting fingers (interquartile range: hand motion, 0.05) in the worse-seeing eye and 0.50 (interquartile range: 0.15, 0.80) in the better-seeing eye.

The age difference between AQP4-ON and ION groups was not significant (mean [SD]: 38.61 [13.72] years vs. 39.07 [16.16] years, *p* = 0.908). The AQP4-ON group had a higher proportion of females than the ION group (82.1% vs. 55.2%, *p* = 0.029) and more recurrence events (median [range]: 0 [0, 4] times vs. 0 [0, 3] times, *p* = 0.014). Compared to the ION group, the AQP4-ON group did not show a significant difference in visual acuity in the better-seeing eye (median [interquartile range]: 0.46 [0.10, 0.94] logMAR vs. 0.30 [0.10, 0.60] logMAR, *p* = 0.242) but had worse visual acuity in the worse-seeing eye (median [interquartile range]: 2.30 [1.85, 2.68] logMAR vs. 1.85 [1.30, 2.30] logMAR, *p* = 0.009). There was no significant difference in the proportion of bilateral severe visual impairment between the AQP4-ON and ION groups (25.0% vs. 13.8%, *p* = 0.462; Table 1).

**Table 1:** Demographic and clinical characteristics of patients with AQP4-ON and idiopathic ON.

Characteristics	Overall (*n* = 57)	AQP4-ON (*n* = 28)	Idiopathic ON (*n* = 29)	*p* value
Age, mean (SD), y	38.84 (14.88)	38.61 (13.72)	39.07 (16.16)	0.908^*^
Female sex, No. (%)	39 (68.4)	23 (82.1)	16 (55.2)	**0.029** ^†^
Number of recurrence events, median [range]	0 [0, 4]	0 [0, 4]	0 [0, 3]	**0.014** ^‡^
Visual acuity in worse-seeing eye, median [interquartile range], logMAR	1.85 [1.40, 2.30]	2.30 [1.85, 2.68]	1.85 [1.30, 2.30]	**0.009** ^‡^
Visual acuity in better-seeing eye, median [interquartile range], logMAR	0.30 [0.10, 0.82]	0.46 [0.10, 0.94]	0.30 [0.10, 0.60]	0.242^‡^
Bilateral severe visual impairment, No. (%)	11 (19.3)	7 (25.0)	4 (13.8)	0.462^†^

AQP4-ON: aquaporin-4 antibody-seropositive optic neuritis; ON: optic neuritis; SD, standard deviation; y, years; logMAR, logarithm of the minimum angle of resolution.

* Student’s *t* test.

† Chi-squared test or chi-squared test with Yates’ continuity correction.

‡ Mann–Whitney U Test. Bold values in table indicate *p* values <0.05.

### Vision-related quality of life

**Figure 2:**
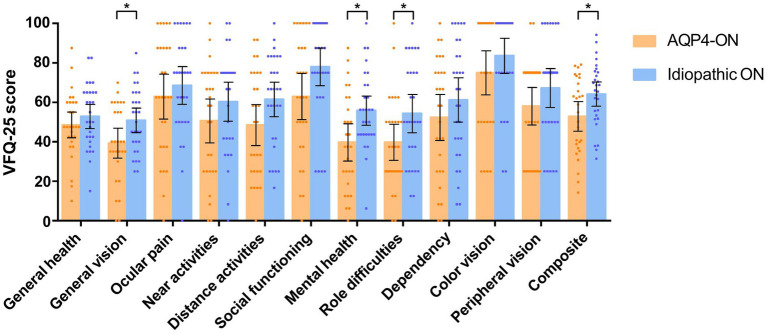
Comparison of scores on the 25-item National Eye Institute Visual Function Questionnaire (VFQ-25) between patients with AQP4-ON and idiopathic ON, analyzed using linear regression models adjusted for sex. Each data spot represents one patient. The results are presented as the mean ± 95% confidence interval (*p < 0.05).

The AQP4-ON group had a lower composite score than the ION group (mean [SD]: 52.92 [19.36] vs. 64.15 [16.14], *p* = 0.021). AQP4-ON was significantly associated with a lower VFQ-25 composite score than ION (Mean difference, −11.65 [95% CI, −21.61 to −1.69]; *p* = 0.023). Additionally, significant associations were found between AQP4-ON and the VFQ-25 scores of general vision (−10.80 [95% CI, −20.86 to −0.74]; *p* = 0.036), mental health (−14.43 [95% CI, −26.66 to −2.19]; *p* = 0.022), and role difficulties (−14.39 [95% CI, −28.16 to −0.62]; *p* = 0.041; Figure 2).

No association was found between AQP4-ON and VFQ-25 scores related to general health, ocular pain, social functioning, dependency, color vision, peripheral vision, near activities, and distance activities (*p* > 0.05 for all variables; Figure 2).

### Depression

**Figure 3:**
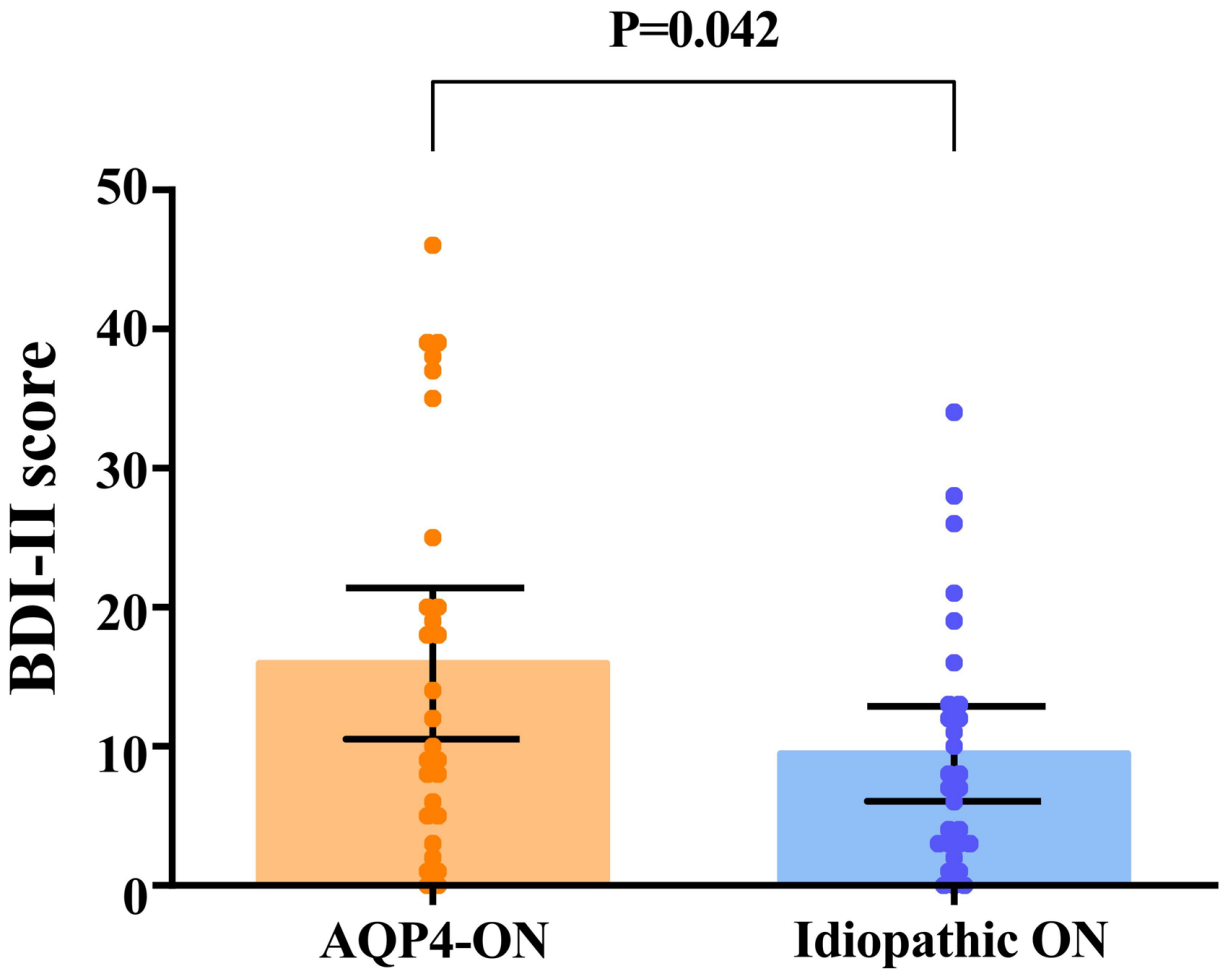
Comparison of scores on the Beck Depression Inventory-II (BDI-II) between patients with AQP4-ON and idiopathic ON, analyzed using an univariate linear regression model. Each data spot represents one patient. The results are presented as the mean ± 95% confidence interval.

None of the participants had a history of mood disorders or were taking mood-stabilizing medications. A higher proportion of AQP4-ON patients had depression than ION patients (13 [46.4%] vs. 6 [20.7%], *p* = 0.039). Patients with AQP4-ON exhibited significantly higher BDI-II scores in comparison to those with ION (Mean difference, 6.48 [95% CI, 0.25 to 12.71]; *p* = 0.042; Figure 3). In the AQP4-ON group, 15 (53.6%) patients had no depression, 4 (14.3%) patients had mild depression, 3 (10.7%) patients had moderate depression, and 6 (21.4%) patients had severe depression. Twenty-three patients (79.3%) in the ION group had no depression, 2 patients (6.9%) had mild depression, 3 patients (10.3%) had moderate depression, and 1 patient (3.5%) had severe depression.

### Correlations between depression and vision-related quality of life and clinical characteristics

**Figure 4:**
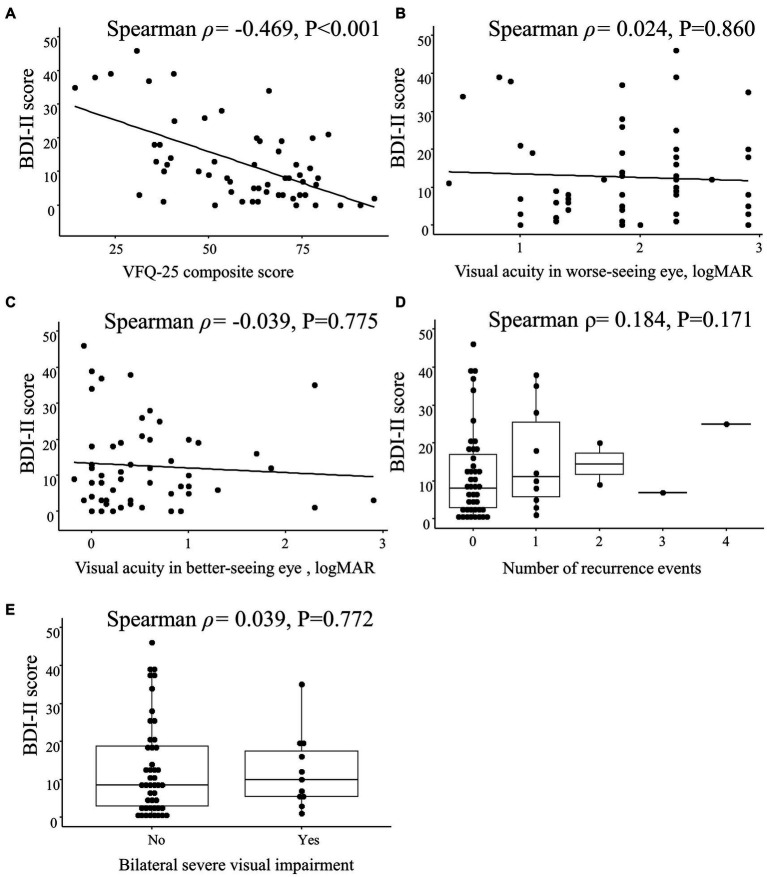
Spearman correlations between Beck Depression Inventory II (BDI-II) score and clinical characteristics in patients with optic neuritis. Scatterplots show the correlations between BDI-II score and **(A)** 25-item National Eye Institute Visual Function Questionnaire (VFQ-25) composite score, **(B)** visual acuity in worse-seeing eye, and **(C)** visual acuity in better-seeing eye. Box plots show the correlation between BDI-II score and **(D)** number of recurrence events and **(E)** bilateral severe visual impairment. Each data spot represents one patient.

The BDI-II score was correlated with the VFQ-25 composite score (Spearman ρ = −0.469; *p* < 0.001) but not with the visual acuity in the worse-seeing eye (Spearman ρ = 0.024; *p* = 0.860) or in the better-seeing eye (Spearman ρ = −0.039; *p* = 0.775), bilateral severe visual impairment (Spearman ρ = 0.039; *p* = 0.772) or the number of recurrence events (Spearman ρ = 0.184; *p* = 0.171; Figure 4).

**Figure 5:**
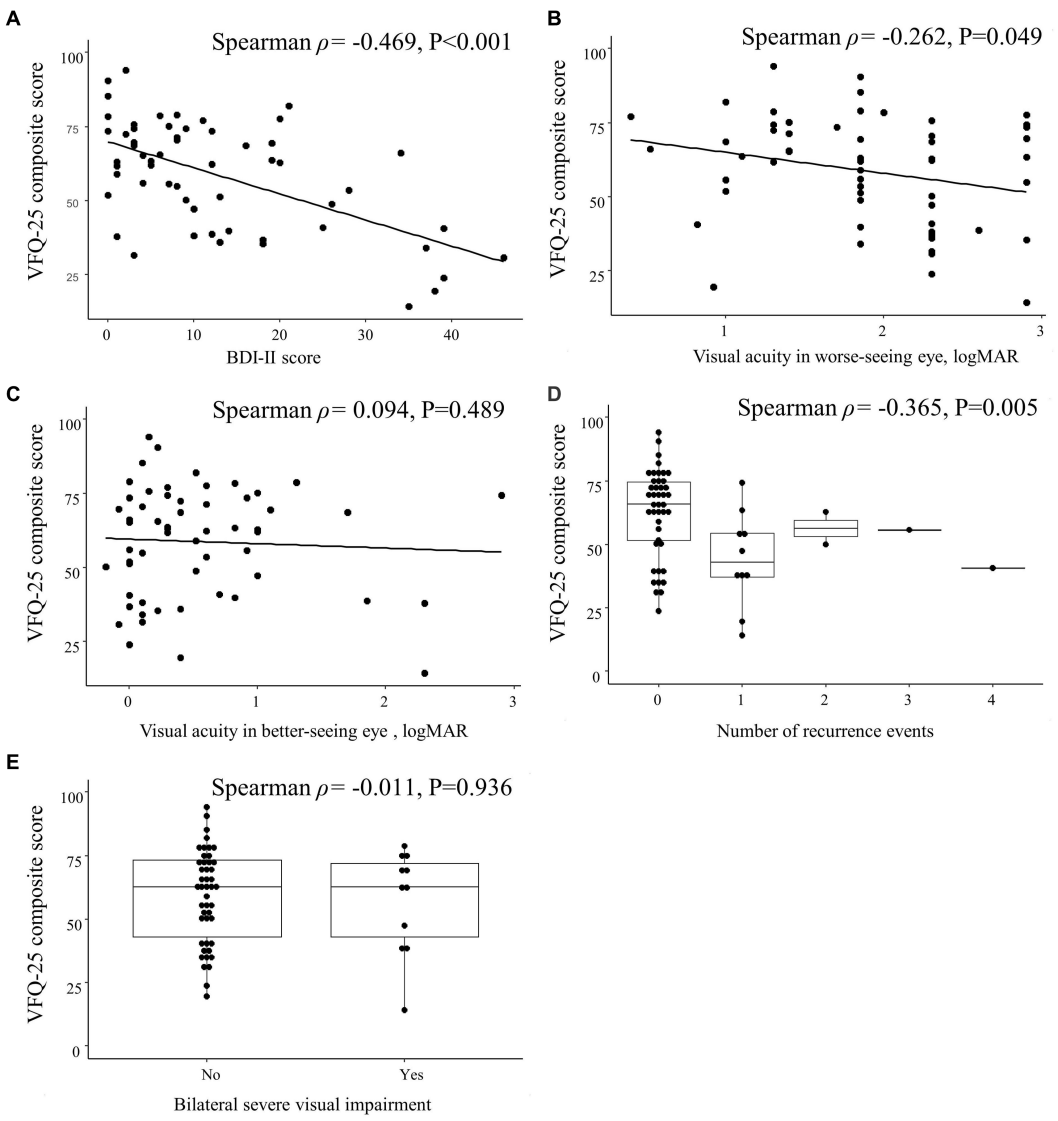
Spearman correlations between 25-item National Eye Institute Visual Function Questionnaire (VFQ-25) composite score and clinical characteristics in patients with optic neuritis. Scatterplots show the correlations between VFQ-25 composite score and **(A)** Beck Depression Inventory II (BDI-II) score, **(B)** visual acuity in worse-seeing eye, and **(C)** visual acuity in better-seeing eye. Box plots show the correlation between VFQ-25 composite score and **(D)** number of recurrence events and **(E)** bilateral severe visual impairment. Each data spot represents one patient.

The VFQ-25 composite score was correlated with the visual acuity in the worse-seeing eye (Spearman ρ = −0.262; *p* = 0.049) and the number of recurrence events (Spearman ρ = −0.365; *p* = 0.005) but not with the visual acuity in the better-seeing eye (Spearman ρ = 0.094; *p* = 0.489) nor with bilateral severe visual impairment (Spearman ρ = −0.011; *p* = 0.936; Figure 5).

## Discussion

This cross-sectional study has demonstrated that the presence of AQP4-ON was associated with a lower VFQ-25 composite score and a higher BDI-II score. The BDI-II score was correlated with the VFQ-25 composite score but not with the visual acuity of either the better-seeing eye or the worse-seeing eye. The VFQ-25 composite score was correlated with visual acuity in the worse-seeing eye but not with the visual acuity in the better-seeing eye.

In patients with AQP4-positive optic neuritis, visual acuity is worse and the retinal nerve fiber layer is thinner than in those with AQP4-negative optic neuritis (9, 14). To the best of our knowledge, there is a lack of specific studies reporting on the impact of aquaporin 4–positive optic neuritis on vision-related quality of life. In the ONTT, patients with unilateral optic neuritis were found to have poorer vision-related QoL than healthy subjects (1). Another study, however, revealed that no patient enrolled in the ONTT was seropositive for AQP4-IgG (15). Jiang et al. reported vision-related QoL using the 39-item National Eye Institute Visual Function Questionnaire (mean composite score: 58.3) in a group of Chinese patients with atypical optic neuritis (16) but did not classify their optic neuritis patients according to glial autoimmune antibodies. It seems that their patients’ vision-related QoL was worse than that of our ION group but better than that of the AQP4-ON group. Also, contrary to Jiang et al.’s findings, the VFQ-25 composite score was associated with visual acuity in the worse-seeing eye but not in the better-seeing eye in our study. In Jiang et al.’s study, however, they found a strong correlation between the VFQ-25 composite score and visual acuity in both the worse-seeing and better-seeing eyes. This difference may be due to the fact that both visual acuity and questionnaires were assessed 3 months after onset in Jiang et al.’s study, whereas our study was performed at admission during the acute attack. In the acute phase, visual acuity loss is more prominent in the worse-seeing eye. Therefore, visual acuity in the worse-seeing eye might account for more vision-related QoL loss during the acute attack.

Schmidt et al. used the 39-item National Eye Institute Visual Function Questionnaire to assess the vision-related QoL for optic neuritis patients and found that patients with NMOSD-ON (mean composite score: 77.4) had lower QoL than patients with multiple sclerosis (MS)-associated optic neuritis (mean composite score: 86.6) (17). In the study by Schmidt et al., the difference in vision-related QoL between MS patients and NMOSD patients was due to the higher incidence of bilateral optic neuritis and more severe damage to retinal structures in the NMOSD group. They found that the retinal nerve fiber layer and ganglion cell layer are usually more severely thinning in NMOSD-ON than in MS-ON, and microcystic macular edema occurs more frequently. Multiple studies have demonstrated the presence of primary retinopathy in patients diagnosed with AQP4-IgG-positive NMOSD using optical coherence tomography (OCT) to assess foveal morphology (18–20). There is reason to suspect that more severe retinal structural changes in AQP4-positive optic neuritis lead to substantial vision-related QoL losses. Furthermore, both NMOSD-ON and MS-ON patients in Schmidt et al.’s study had higher vision-related QoL than the patients in our study. This discrepancy may be due to differences in patient ethnicity; the majority of Smith et al.’s study was Caucasian (93.5%), and our study included all Asian patients. The severity of attacks was found to differ based on race in an international cohort of patients with NMOSD (21).

In our study, 46% of patients with AQP4-ON were considered positive for depression based on the BDI-II. The proportion of depression observed in our study aligns with the findings reported in previous studies conducted on NMOSD patients in Spain (44.3%) (6) and Germany (39.8%) (22). However, our study found a higher proportion compared to studies conducted on patients with other ocular diseases such as glaucoma and dry eye disease, as reported in a systematic review (5). It’s noteworthy that our study, along with the two NMOSD studies, utilized the BDI as a measure of depression, whereas the studies included in the systematic review used a variety of different depression scales, including the BDI. None of these studies conducted professional psychiatric evaluations, so the depressive symptoms observed may be an acute reaction to the illness rather than a depressive disorder. In recent years, the immune inflammation hypothesis has been considered a possible mechanism of depression, by which pathogenesis and pathophysiology process of the disease are influenced by interactions between inflammation pathways and neural circuits and neurotransmitters (4). Elevated levels of TNF-α and IL-6 in the cerebrospinal fluid and brain parenchyma of patients with depression may be associated with enhanced microglial activity and decreased levels of astrocyte and oligodendrocyte markers (23). Although no study has yet to investigate whether AQP4 antibody-positive optic neuritis and depression share a common pathophysiological mechanism, we believe that severe inflammation might be responsible for the high proportion of depression in AQP4 antibody-positive patients.

Our analysis found that the BDI-II score was correlated with the composite score of VFQ-25 but not with the visual acuity in the better-seeing or worse-seeing eye or with bilateral severe visual impairment. This is similar to previous findings in glaucoma (24), and may be because visual acuity represents only one aspect of visual function. It is therefore appropriate to evaluate patients who have lower vision-related QoL without a decrease in objective vision acuity for psychological disorders as well. This finding could help ophthalmologists identify patients with psychological disorders early.

There are some limitations to this study. First, considering the limited sample size and exploratory nature of this study, our study did not adjust for all potential confounding variables, including visual acuity and number of recurrent events, nor did we conduct any corrections for multiple comparisons. We intend to increase the sample size in future studies to facilitate a more comprehensive analysis. Second, the presence of depression in this study was determined exclusively using the BDI-II questionnaire, without a professional psychiatric evaluation. Consequently, it remains undetermined whether the observed depressive symptoms represent an acute reaction to sudden vision loss or a mood disorder. Third, the patients included in our study did not undergo serum myelin oligodendrocyte glycoprotein (MOG) antibody testing, so there may have been MOG antibody-positive patients in the ION group. However, MOG-ON is relatively rare in Chinese patients with ON, accounting for approximately 22% (8), particularly in our center, where it accounts for only 6% in a recent cohort (unpublished data). Fourth, due to the limited availability of OCT devices and high cost of OCT examinations in our setting, OCT data could not be collected for this study. Future research may consider examining the relationship between retinal neurodegeneration parameters (such as retinal nerve fiber layer thickness or ganglion cell layer thickness) and vision-related QoL and depression, in a more extensive cohort of patients. Last, these patients were recruited from tertiary centers and may be biased toward more severe diseases.

## Conclusion

In summary, AQP4-positive optic neuritis was found to be significantly associated with impaired vision-related quality of life and an increased occurrence of depressive symptoms. Clinicians should assess vision-related QoL and depressive symptoms in patients with AQP4-positive optic neuritis to facilitate patient-centered care.

## Data availability statement

The raw data supporting the conclusions of this article will be made available by the authors, without undue reservation.

## Ethics statement

The studies involving humans were approved by Medical Ethics Committee of the First Affiliated Hospital of Guangxi Medical University. The studies were conducted in accordance with the local legislation and institutional requirements. The participants provided their written informed consent to participate in this study.

## Author contributions

RS: Writing – original draft, Formal analysis, Visualization. WH: Writing – original draft, Data curation, Formal analysis, Investigation, Visualization. XT: Writing – review and editing, Investigation. JY: Writing – review and editing. YH: Writing – review and editing, Investigation. YC: Writing – review and editing, Funding acquisition, Investigation. MZ: Writing – review and editing, Investigation. QH: Writing – review and editing. YD: Writing – review and editing, Writing – original draft, Conceptualization, Data curation, Funding acquisition, Investigation, Methodology, Supervision.

## Funding

The author(s) declare financial support was received for the research, authorship, and/or publication of this article. This work was supported by the National Natural Science Foundation of China (No. 82260202), the Scientific Research Project of Guangxi Health Commission (No. Z20182008), the National College Students Innovation and Entrepreneurship Training program project (202010598003), and “Medical Excellence Award” funded by the Creative Research Development grant from the First Affiliated Hospital of Guangxi Medical University.

## Conflict of interest

The authors declare that the research was conducted in the absence of any commercial or financial relationships that could be construed as a potential conflict of interest.

## Publisher’s note

All claims expressed in this article are solely those of the authors and do not necessarily represent those of their affiliated organizations, or those of the publisher, the editors and the reviewers. Any product that may be evaluated in this article, or claim that may be made by its manufacturer, is not guaranteed or endorsed by the publisher.
