# Supplementary material for: Association of aquaporin-4 antibody-seropositive optic neuritis with vision-related quality of life and depression
Source: Front Neurol. 2023 Sep 29;14:1265170. doi: 10.3389/fneur.2023.1265170 (PMC10575616; doi:10.3389/fneur.2023.1265170)
Supplement: Supplementary file 1 [file Table_1.DOCX]

Supplemental Table. Distribution of patients across the four study sites.

| Site | AQP4-ON  No. (%) | idiopathic ON  No. (%) | Total |
| --- | --- | --- | --- |
| 1 | 23 (82.14) | 23 (79.31) | 46 |
| 2 | 4 (14.29) | 4 (13.79) | 8 |
| 3 | 1 (3.57) | 1 (3.45) | 2 |
| 4 | 0 | 1 (3.45) | 1 |
| Total | 28 | 29 | 57 |

AQP4-ON: aquaporin-4 antibody-seropositive optic neuritis; ON: optic neuritis.

1. The First Affiliated Hospital of Guangxi Medical University, Nanning, China.

2. Wuzhou Gongren Hospital, Wuzhou, China.

3. Wuming Hospital of Guangxi Medical University, Nanning, China.

4. The Second Affiliated Hospital of Guangxi Medical University, Nanning, China.
